# Low antiretroviral therapy uptake and low early retention among pregnant women who tested positive for human immunodeficiency virus in informal health centers in urban and semi-rural settings in Cameroon: a prospective cohort study

**DOI:** 10.3389/fpubh.2023.1188749

**Published:** 2023-07-26

**Authors:** Audrey Amboua Schouame Onambele, Francis Yuya, Arielle Andtoungou Schouame, Sylvie Kwedi Nolna, Antoine Socpa

**Affiliations:** ^1^Department of Public Health, School of Health Sciences, Catholic University of Central Africa, Yaoundé, Cameroon; ^2^Institut de Recherche pour le Développement France, Yaoundé, Cameroon; ^3^Epicentre, Niamey, Niger; ^4^Department of Disease, Epidemics, and Pandemics Control, Ministry of Public Health, Yaoundé, Cameroon; ^5^Capacity for Leadership Excellence and Research (CLEAR), Yaoundé, Cameroon; ^6^Department of Public Health, Faculty of Medicine and Biomedical Sciences, University of Yaoundé I, Yaoundé, Cameroon; ^7^Center for Applied Social Sciences, Research and Training (CASS-RT), Yaoundé, Cameroon

**Keywords:** HIV, PMTCT, informal health centers, pregnant women, ART naïves

## Abstract

**Introduction:**

Despite the efforts of Cameroon’s Ministry of Public Health against informal health centers (IHCs) because of their illegitimacy, the number of IHCs is increasing in Cameroon. Most of these IHCs have antenatal care services and screen pregnant women for HIV. However, nothing is known about the subsequent outcomes of those who tested positive for HIV. This study aimed to assess the initiation of antiretroviral therapy (ART) in ART-naïve pregnant women screened HIV positive in IHCs within three months of diagnosis and their ART retention at three months post-initiation. In addition, we sought to identify the factors associated with ART non-initiation in this population.

**Methods:**

May 01, 2019 to August 31, 2020, we carried out a prospective cohort study of ART-naïve pregnant women who attended their first antenatal care visit and screened HIV positive at IHCs in the cities of Douala and Ebolowa in Cameroon. Standardized questionnaires were used to interview consenting participants at three points: the day of the delivery of the antenatal HIV test result, three months later, and three months after ART initiation. The data collected were entered into KoboCollect and analyzed using SPSS V23.0 software. The Chi-square test was used to compare proportions, Kaplan Meier techniques and Cox proportional hazards regression was used to estimate retention in ART and identify factors associated with ART non-retention, respectively.

**Results and discussion:**

A total of 85 ART-naïve pregnant women living with HIV were enrolled in the study. The median age and gestational age at the first antenatal care visit were 29 years (interquartile range (IQR), 2333.5) and 28weeks of amenorrhea (IQR, 2032), respectively. Only 34% (29/85) initiated ART, and 65.5% (19/29) of the initiators were retained in ART three months later. Lack of perceived self-efficacy to initiate ART (adjust Hazard Ratio = 5.57, 90% CI: 1.29 to 24.06), increased the probability of not be retaining in ART by any time during three months post initiation. Given the low ART uptake and the low retention in care among pregnant women living with HIV screened in IHCs, PMTCT policies in Cameroon should pay greater attention to this population, to facilitate their continuum of PMTCT care.

## 1. Introduction

Mother-to-child transmission of the human immunodeficiency virus (HIV) is the leading cause of pediatric HIV (1–5). Globally, 150,000 new infections were registered in children in 2020, 87% of which occurred in sub-Saharan Africa (6). In the same year, 4,500 new infections due to vertical transmission were reported in Cameroon. Among these new infections, 60% were due to the non-enrollment of women living with HIV on antiretroviral therapy (ART) during pregnancy or breastfeeding, and 23% to their non-retention in ART (7).

Preventing HIV infection in children begins by controlling the infection in mothers living with HIV. Thus, the screening of pregnant women, enrollment of those living with HIV in care, and their retention in the continuum of care are essential for the success of programs aimed at preventing mother-to-child transmission of HIV (PMTCT) (8, 9).

To improve the effectiveness of PMTCT programs, Cameroon, following international recommendations, successively moved from Option B+ in 2012 (whereby all pregnant and breastfeeding women were eligible for lifelong ART as soon as they were diagnosed with HIV, regardless of the CD4+ cell count or WHO clinical stage) to the test and treat strategy since 2016 in which ART eligibility is no longer limited to pregnant and breastfeeding women but expanded to anyone living with HIV (10). Nevertheless, the success of PMTCT programs depends on the enrollment in ART and the retention of mothers in the continuum of PMTCT care (11), which begins with the offer of antenatal HIV screening at the first antenatal care (ANC) visit, which is the gateway to PMTCT in health facilities (12, 13).

In 2017, the National Committee for the fight against Acquired Immune Deficiency Syndrome (AIDS) in Cameroon suspected the high attendance of informal health facilities in urban areas as the cause of weak performances recorded by the PMTCT program, especially regarding the low rate of attendance at ANC and childbirth services (14). Indeed, the informal health sector has been growing in Cameroon for years and is overwhelmed by a plethora of informal care providers (15–18). In 2007, the third Cameroonian population survey showed that slightly more than 30% of Cameroonians sought care in the informal health sector (19). A little over a decade later, a study highlighted the rise of informal health centers (IHCs) in Cameroon, emphasizing that these were neither listed nor controlled by public authorities (20). In April 2017, more than 3,000 IHCs were counted throughout the national territory (21).

Although statistics on patients who use IHCs services are not available, we could, because of this growth of IHCs, deduce that they attend to a significant number of patients.

IHCs generally offer a wide range of services, including ANC services, the starting lever for PMTCT. As a result, they could represent an important Actor to consider in the fight against mother-to-child transmission of HIV in Cameroon. However, nothing is known about the future of pregnant women diagnosed with HIV infection in these IHCs.

The objective of this study was to investigate the initiation of ART in ART-naïve pregnant women living with HIV (PWLHIV) tested in IHCs and their retention 3 months after initiation. In addition, this study sought to determine factors associated with ART non-retention among this population in two cities in Cameroon: Douala and Ebolowa.

## 2. Materials and methods

### 2.1. Study design

From May 01, 2019 to August 31, 2020, we conducted a prospective cohort study in Douala and Ebolowa, two cities in Cameroon.

### 2.2. Study setting

Douala and Ebolowa are the capitals of the Littoral and South regions in Cameroon, respectively. Douala is urban, and Ebolowa is semi-rural. In 2019, the HIV seroprevalence rates among newly screened pregnant women in the littoral and south regions were 1.9% and 3%, which were above the national average of 1.7% (22).

Cameroon’s health system is divided into three levels. A central, an intermediate, and a peripheral level. In terms of health facilities, the central level is made up of general hospitals, central and similar hospitals, and university teaching hospitals. In addition, the second level is made up of regional and similar hospitals. Furthermore, the peripheral level is constituted of, in hierarchical order, district hospitals, clinics, subdivisional medical centers, integrated health centers, and care units (23). The peripheral level is closest to the population and is managed by a Health District Service.

Overall, public health facilities at all levels are facing many dysfunctions that include poor reception, extortion and diversion of patients, delay in their care, and lack of compassion for patients in distress (24, 25). These dysfunctions have contributed to the emergence of IHCs in Cameroon. IHCs are perceived by the population as more patient-friendly due to shorter waiting times for care, low prices, proximity, and better reception (20).

#### 2.2.1. Definition of informal health centers

We defined an IHC as any secular private health facility with a fixed location, without an official government-issued act of creation or operation and without Physicians.

#### 2.2.2. Description of informal health centers

##### 2.2.2.1. Health staff

Of the 183 staff involved in maternal and child health (MCH) in IHCs, 56% (103/183) were nurse assistants, 27% (49/183) were nurses, 10% (19/183) were laboratory technicians, and 7% (12/183) had no documented training and were trained onsite by colleagues. Apart from the latter with no official health training, all the others had received conventional training in health but were generally unable to integrate into the formal health sector. However, it should be clarified that 28% (51/183) of those health staff were civil servants or former civil servants of the Ministry of Public Health. No more than four staff were found per IHCs.

##### 2.2.2.2. Premises

The IHCs were small premises subdivided into three or four boxes. Some came from the division of a residence (parts of the residence were used to make the IHC).

For IHCs that dispensed ART, a few boxes were placed on a shelf in the room. The stock of antiretrovirals (ARVs) per IHC did not exceed five boxes, and no ARV stock tracking sheet was available.

##### 2.2.2.3. Collection of health information

The tracing of ANC, delivery, and ART dispensing registers was not harmonized. Several registers could be used for the same year, and the years could overlap within the same register without any chronological order.

However, in almost a quarter of the IHCs that dispensed ART, there were harmonized ART-dispensing registers approved by the Ministry of Public Health, while the others used hand-drawn records.

The registration of patients in ANC was not systematic, and patient information was not always complete. Therefore, in order to obtain the result of the HIV screening test, it was sometimes necessary to use the laboratory register.

##### 2.2.2.4. Management of PWLHIV

Within the IHCs, we found two types of management of women screened as HIV-positive during ANC. The first was to refer women ro HIV care centers. In this case, the reference was mostly verbal (healthcare providers told PWLHIV where they could get ART) and sometimes written in the ANC register, but no referral document was issued to the woman.

Secondly, the IHCs supplied ART to PWLHIV, and ART was integrated into the ANC. In this case, women were supplied with ART monthly although this supply was limited in time, usually with the birth of the child (2 months post-partum). This means that even in this management type, the woman will subsequently be referred with her child to formal PMTCT care centers for the continuum of care.

##### 2.2.2.5. ART supply

In Cameroon, health facilities at the peripheral level are supplied with ARV treatments by health districts in their area. HIV care has been decentralized to the level of integrated health centers managed by nurses. However, IHCs claimed to receive ARV treatment from public health structures (health districts and public health facilities).

### 2.3. Selection of informal health centers

We used the health map of Douala and Ebolowa to select IHCs. Cameroon’s health map divides each city into health districts. Douala has nine health districts: Boko, Japoma, Nylon, Bangue, Deido, Cité des Palmiers, Bonassama, Newbell, and Logbaba.

We randomly selected seven of the nine health districts: Nylon, Bangue, Deido, Cité des Palmiers, Bonassama, Newbell, and Logbaba. Since Ebolowa has only one healthdistrict, the entire district was retained. After selecting health districts in the two research locations, field workers recruited for the study had a starting point in each health district. This starting point was the benchmark for the census of the IHCs within the health district.

### 2.4. Participants

The study population consisted of pregnant women at any stage of the pregnancy who tested positive for HIV in IHCs at their first ANC for the considered pregnancy and were naïve to ART at this first ANC visit. Among the 94 women enrolled in the main study, 85 were ART-naïve at their first ANC and were retained for the present study.

#### 2.4.1. Recruitment of participants

**Figure 1:**
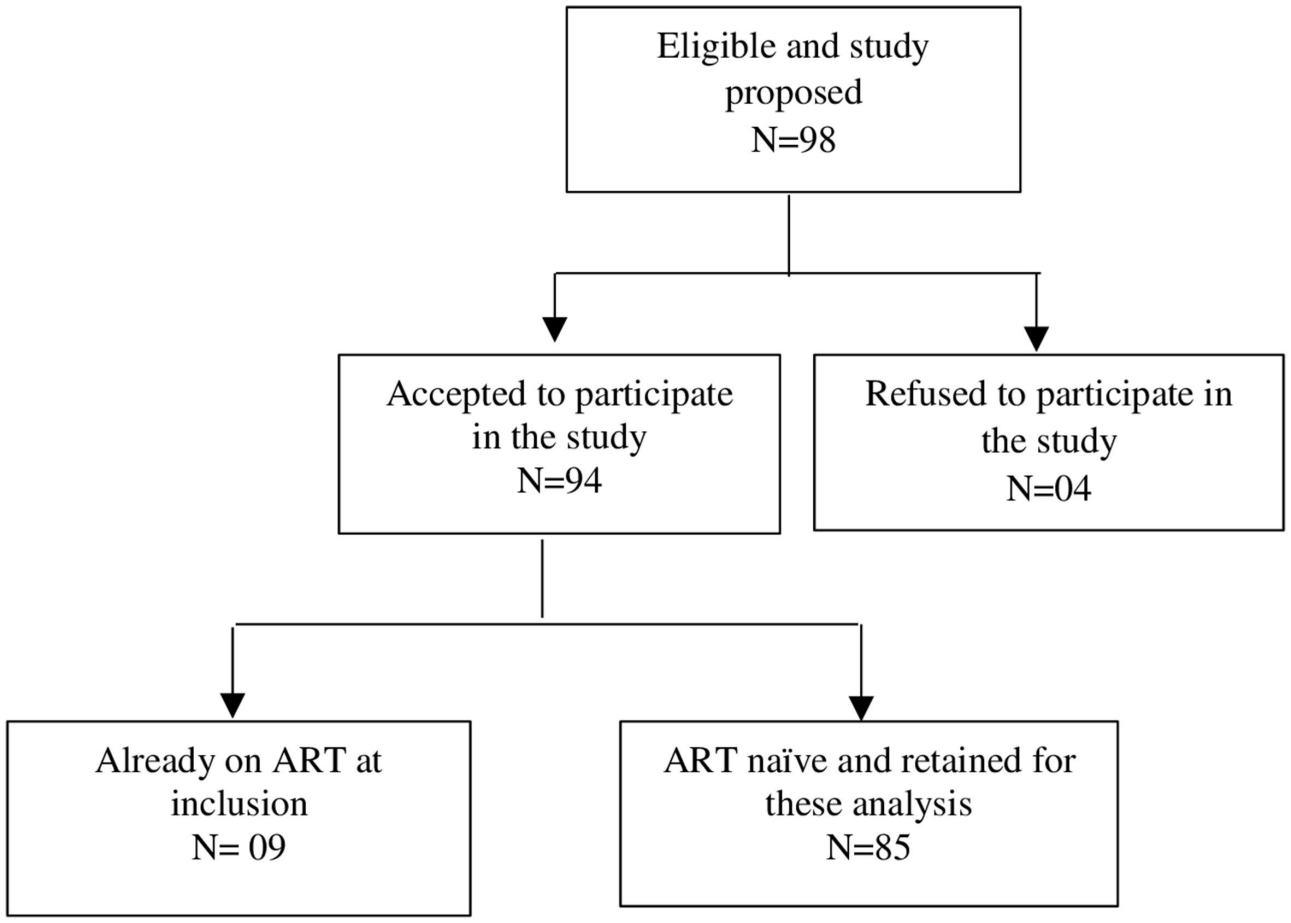
Flow chart of participant enrollment in the study.

Nurses or nurse assistants in charge of ANC services explained and proposed the study to pregnant women who tested positive for HIV between May 01, 2019 to April 30, 2020. Those who agreed signed the written informed consent for study participation and were enrolled in the study. Of the 98 women living with HIV to whom the study was proposed, 94 agreed to participate. Among them, 85 were naïve to any ARV treatment and were finally retained for this analysis (Figure 1).

### 2.5. Definition of outcomes

#### 2.5.1. ART uptake/ART initiation

This corresponds to the number of pregnant women who tested positive for HIV during ANC in IHCs and started ART, either in the IHCs or following a referral to an ART Center. In this study, we considered those who started ART within 3 months of their HIV diagnosis.

#### 2.5.2. ART non-initiation

This corresponds to pregnant women who tested positive for HIV during ANC in IHCs, who were alive but were not initiated on ART within 3 months of their HIV diagnosis.

#### 2.5.3. ART retention

In this study, retention in ART care was defined as the number of live participants who continued to collect their ART either in the IHCs or in any other ART clinic during the first 3 months after their ART initiation, with a 14 days window in the third month.

Those who disengaged from care within the 3 months of ART initiation or were lost to follow-up by the time of evaluation were considered not retained.

### 2.6. Data

#### 2.6.1. Source of data

The data were collected as part of a larger study entitled “ECIP PTME,” which assessed PMTCT in IHCs. It included 94 pregnant women who tested HIV positive during their first antenatal consultation for the pregnancy considered in the study. The study was conducted in Douala and Ebolowa in 110 IHCs.

#### 2.6.2. Data collection

Three standardized questionnaires were developed to interview women: T0, T1, and T2. The T0 captured data on sociodemographics, parity, relationship duration with an intimate partner, history of HIV testing, intention to share HIV results with a partner, and self-efficacy perception to initiate ART. The T1 questionnaire was used to collect information on the initiation of ART, reasons for non-initiation, and place of ART supply. Lastly, T2 assessed retention at 3 months post ART initiation and reasons for non-retention.

Participants were interviewed at three time points: on inclusion—the day of the delivery of the antenatal HIV test result (T0); 3 months after the delivery of the antenatal HIV test result (T1); and 3 months after ART initiation (T2).

The T1 and T2 were administered on the same day for participants whose dates of ART initiation and delivery of HIV results were similar, as well as for those who missed the T1 but were caught up on their T2 interview visit.

The questionnaires were administered by nurses or nurse assistants who included participants in the study, either face-to-face or by phone.

For answers given by the women during the administration of the questionnaires, such as the initiation of ART and retention in care, we compared the data from the ART dispensing registers and monitoring sheets. Meanwhile, women who were referred to ART dispensing centers provided us with their treatment identifiers. Therefore, we could check their information either directly on their prescription renewal sheets or in the ART dispensing registers of the structure in which they were referred to collect their ART.

The women received a reminder call if they were not seen or could not be reached by phone 2 days after the scheduled interview date. If they did not honor the new appointment within a week, they were re-called every 2 weeks until they were finally available within the study deadline. All collected data were entered into KoboCollect.

#### 2.6.3. Data analysis

The descriptive statistics performed included frequencies and percentages for categorical variables and median and interquartile ranges (IQRs) for continuous variables. Proportions were used to calculate ART uptake and retention. The chi-square test was used to compare the proportions of initiation of ART and retention among PWLHIV between women enrolled in IHCs with no ART and those enrolled in IHCs where ANC integrated ART (*p* < 5%).

Estimates of retention in ART were determined using Kaplan–Meier. Cox proportional hazards regression was used to identify associated factors to ART non-retention. Covariables that were significant in the bivariate analysis (*p* < 10%) were adjusted in the final multivariate model at a level of 10% significance. The covariates used for this purpose were PMTCT management, age group, study level, marital status, income-generating activity, relationship duration, pregnancy willingness, awareness of HIV status of their male partner, knowledge of MTCT of HIV before ANC, and perceived self-efficacy to initiate ART. Analyses were performed in SPSS Statistics software version 23.0.

### 2.7. Ethics statement

This study was approved by the Cameroon National Ethics Committee for Research in Human Health (Ethical clearance no. 2019/03/1155/CE/CNERSH/SP) and by the Cameroonian Ministry of Public Health (Administrative authorization no. 631-15.19) before the implementation of the study. In addition, informed and signed consents from participants were also collected.

## 3. Results

### 3.1. Description of the population

A total of 85 ART-naïve PWLHIV were considered for this study. The median age at enrolment in the study was 29 years (IQR, 23–33.5), and the median gestational age at the first ANC was 28 weeks (IQR, 20–32). Approximately 75.3% (64/85) of participants were enrolled in IHCs without ART that referred to PMTCT centers for further follow-up. Meanwhile, approximately 25% (21/85) of the participants were enrolled in IHCs with ANC integrating ART. More than half of PWLHIV 63.5% (54/85) had a secondary education level, and 61.1% (33/54) of them interrupted their studies in the first cycle of secondary school. Only 29.4% (25/85) of our participants had an income-generating activity, and this was mainly (23/25) within the informal sector. Regarding marital status, 64.7% (55/85) were married or cohabiting. Among their male partners, 84.7% (72/85) had an income-generating activity, mainly within the informal sector (59/72). Moreover, 75.3% (64/85) of the participants were multiparous, and only 12.9% (11/85) had planned their pregnancy. However, pregnancy was desirable in 64.7% (55/85) of the participants. Approximately 21.2%% (18/85) of the participants already knew their HIV status before the current pregnancy, and 83.3% (15/18) discovered their status during a previous pregnancy. In addition, 10.5% (9/85) of participants were aware of the HIV status of their male partners, and 63.5% (54/85) were informed of the vertical transmission of HIV (see Table 1).

**Table 1:** Characteristics of participants.

	Frequency	Percentage
Town *(n = 85)*		
Douala	58	68.2
Ebolowa	27	31.8
Type of IHC *(n = 85)*		
ANC integrating ART	21	24.7
Refer for ART	64	75.3
Age group *(n = 85)*		
15–24	27	31.8
25–34	41	48.2
35–44	17	20
*Weeks of amenorrhea (n = 85)*		
<16	10	11.8
16–28	41	48.2
˃28	34	40
*Study level (n = 85)*		
Primary	21	24.7
Secondary	54	63.5
University	10	11.8
*Income-generating activity (n = 85)*		
Yes	25	29.4
No	60	70.6
*Marital status (n = 85)*		
Single	30	35.3
Married/cohabiting	55	64.7
*Income-generating activity of partner (n = 85)*		
Yes	72	84.7
No	13	15.3
*Relationship duration (n = 85)*		
<2 years	56	65.9
˃2 years	29	34.1
*Parity (n = 166)*		
Primiparous	21	24.7
Multiparous	64	75.3
*Planned pregnancy (n = 85)*		
Yes	11	12.9
No	74	87.1
*Wanted pregnancy (n = 85)*		
Yes	55	64.7
No	30	35.3
*Awareness of HIV status of their male partner (n = 85)*		
Yes	9	10.6
No	76	89.4
*Awareness of vertical transmission of HIV (n = 85)*		
Yes	54	63.5
No	31	36.5
*Perceived capacity to initiate ART (n = 166)*		
Yes	23	27.1
No	62	72.9

### 3.2. Art initiation and retention in ART 3 months post initiation

#### 3.2.1. ART initiation

Nearly 34% (29/85) of the participants initiated ART. The median time between the delivery of the HIV results and initiation of ART was 7 days (IQR, 0–18). For women who initiated ART in IHCs that integrated ART in ANC, the median time to initiate ART was 4 days (IQR, 0–12), while this was longer for women who were referred, 16 days (IQR, 7–37). Moreover, among participants who attended their first ANC in IHCs with ART, 47% (8/17) and 82.3% (14/17) initiated ART the day they withdrew their HIV result and within 2 weeks of the receipt, respectively. For those who were referred to formal ART centers, 8.3% (1/12) initiated ART on the same day they had their HIV results, and 50% (6/12) initiated within 2 weeks. Therefore, approximately 69% (20/29) of women initiated ART within 2 weeks of their diagnosis.

After the delivery of the antenatal HIV test result, 81.2% (52/64) of participants who were referred for the continuum of care and 19% (4/21) screened in IHCs that integrated ART in ANC were not initiated on ART, and this difference in proportions was significant (*p* < 0.001), Table 2.

**Table 2:** Distribution of ART initiation and retention among PWLHIV according to the availability of ART in informal health centers.

	PWLHIV enrolled in IHCs that refer for ART (*N* = 64)	PWLHIV enrolled in IHCs that integrated ART in ANC (*N* = 21)	Total (*N* = 85)	*p*-value
*ART initiation*				
Yes	12 (18.8%)	17 (81%)	29 (34.1%)	<0.001
No	52 (81.2%)	4 (19%)	56 (65.9%)	

	PWLHIV enrolled in IHCs that refer for ART (*N* = 12)	PWLHIV enrolled in IHCs that integrated ART in ANC (*N* = 17)	Total (*N* = 29)	*p*-value
*ART retention 3 months post initiation*				
Yes	8 (66.7%)	11 (64.7%)	19 (65.5%)	0.61
No	4 (33.3%)	6 (35.3%)	10 (34.5%)	

The main reasons reported by the 56 women who did not initiate ART were in descending order: fear of reception in the referral structure (22/56), fear of stigmatization by the partner or intimate partner violence (14/56), denial of illness (12/56), poor reception in the referral structure (6/56), and fear of stigmatization by family members (2/56).

#### 3.2.2. ART retention 3 months post ART initiation

Regarding ART retention at 3 months, 65.5% (19/29) of women who initiated ART were retained in care. The retention was not statistically different (*p* = 0.61) between women who were enrolled in IHCs that integrated ART in ANC and those who were referred for ART (Table 2).

**Figure 2:**
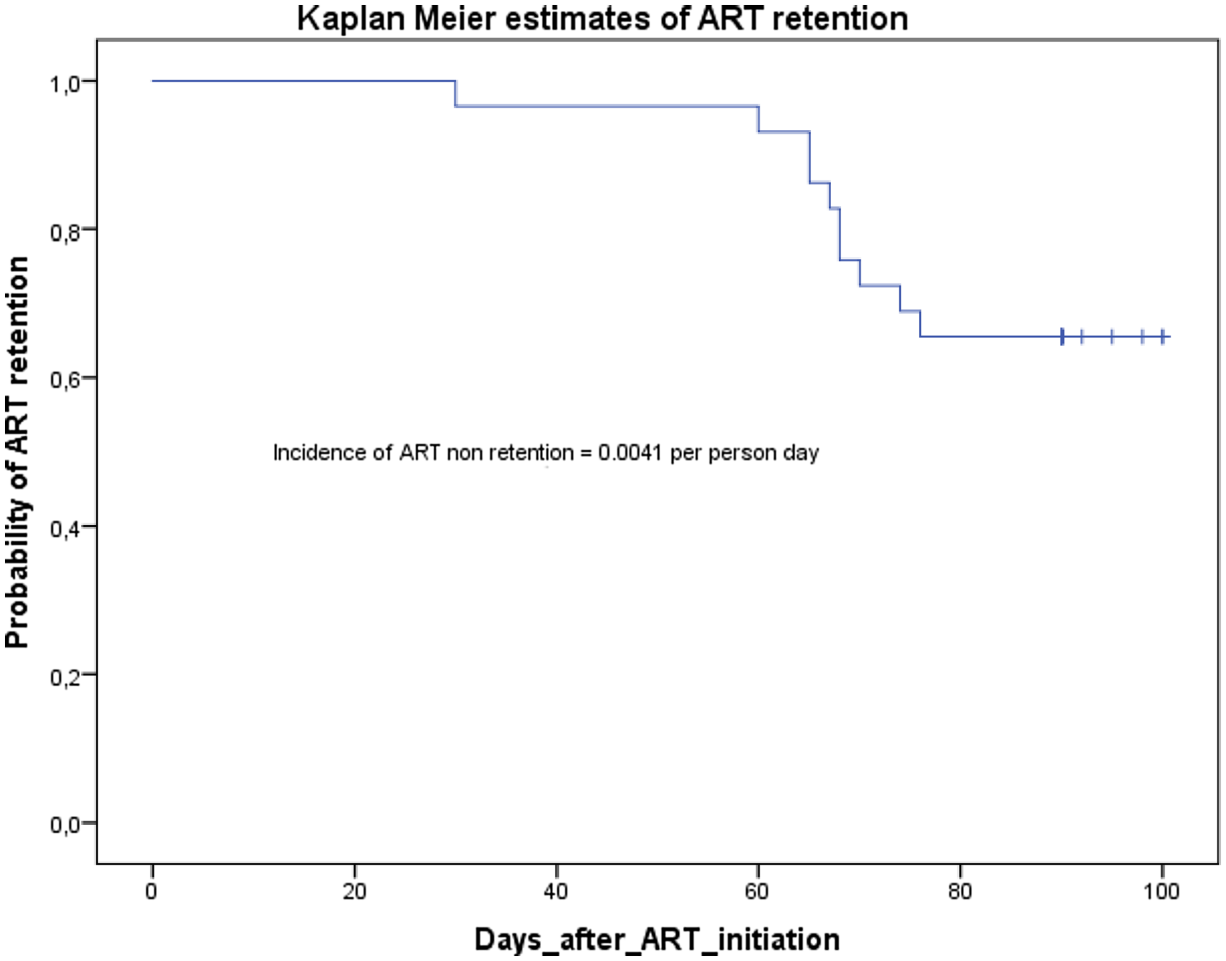
Kaplan–Meier estimates of ART retention at 3 months (90 days) post ART initiation.

As we can see in the Kaplan–Meier curve in Figure 2, all the participants were retained at month one (30 days). At months two (60 days), the probability of ART retention was 0.93; and this probability dropped to 0.65 at months three (90 days, with 14 days window). The incidence rate of non-retention in care was 0.0041 per person day.

All the participants who were not retained were disengaged from care. No lost to follow-up was registered since we had information on all not retained participants at the time of the study endpoint. The main reasons stated by the 10 women who were not retained in ART are in descending order: poor reception in the referral structure (3/10), fear of stigmatization by the partner or intimate partner violence (2/10), denial of illness (2/10), fear of side effects (1/10), unavailability of treatments (1/10), and fear of COVID-19 (1/10).

### 3.3. Factors associated with ART non-retention

#### 3.3.1. Bivariate analysis

In the bivariate analysis, non-initiation of ART was associated with age group, activity, duration of the relationship, the state of pregnancy willingness, the perceived self-capacity to initiate ART, and the awareness of MTCT of HIV before the ANC.

Thus, participants aged less than 25 years had a higher probability of not being retained in ART relative to older participants [hazard ratio (HR) = 3.83, 90% CI: 1.02 to 14.34, *p* = 0.09]. Participants without an income-generating activity had approximately four times an increased probability of not being retained in ART (HR = 3.90, 90% CI: 1.06 to 14.34, *p* = 0.08). Moreover, this probability was also increased in participants whose relationship duration was less than 2 years (HR = 3.26, 90% CI: 1.12 to 9.45, *p* = 0.06).

In addition, ignorance of MTCT of HIV (HR = 5.17, 90% CI: 1.59 to 16.82, *p* = 0.02), carrying an unwilling pregnancy (HR = 3.83, 90% CI: 1.02 to 14.34, *p* = 0.09), and lack of self-perceived capacity to initiate ART after HIV results (HR = 6.20, 90% CI: 2.11 to 18.20, *p* = 0.005) also increased this probability.

Nonetheless, in bivariate analysis, the type of setting (urban or semi-rural) was not associated with ART non-retention. Whether a woman was enrolled in an IHC in Douala (urban setting) or Ebolowa (semi-rural setting) did not affect her probability of not being retained in ART.

#### 3.3.2. Multivariable analysis

In the multivariable-adjusted model, only the perceived self-efficacy to initiate ART after HIV diagnosis was significantly associated with non-retention in ART at 3 months of follow-up (Table 3). Indeed, a lack of perceived self-efficacy to initiate ART after HIV diagnosis increased the probability of not being retained in ART [adjust hazard ratio (aHR) = 5.57, 90% CI: 1.29 to 24.06, *p* = 0.05].

**Table 3:** Factors associated with ART non-retention in pregnant women screened as HIV positive in informal health centers.

	Bivariate analysis		Multivariable analysis	
	HR (90% CI)	*p*	aHR (90% CI)	*p*
*Town*				
Douala	1			
Ebolowa	1.13 (0.39–3.29)	0.84		
*Type of IHC*				
ANC integrating ART	1			
Refer for ART	0.96 (0.33–2.79)	0.95		
*Age group*				
<25	3.83 (1.02–14.34)	0.09	1.50 (0.21–10.41)	0.72
≥25	1		1	
*Study level*				
Primary	1.04 (0.18–5.89)	0.97		
Secondary & more	1			
*Income activity*				
Yes	1		1	
No	3.90 (1.06–14.34)	0.08	1.24 (0.26–5.92)	0.81
*Marital status*				
Single	1.23 (0.33–4.52)	0.79		
Married/ cohabiting	1			
*Aware of the HIV status of their male partner*				
Yes	1			
No	1.46 (0.39–5.38)	0.62		
*Relationship duration*				
<2 years	3.26 (1.12–9.45)	0.06	3.01 (0.71–12.59)	0.20
≥2 years	1		1	
*Pregnancy willingness*				
Yes	1		1	
No	3.83 (1.09–14.34)	0.09	1.50 (0.21–10.41)	0.72
*Perceived capacity to initiate ART*				
Yes	1		1	
No	6.20 (2.11–18.20)	0.005	5.57 (1.29–24.06)	0.05
*Aware of MTCT of HIV before ANC*				
Yes	1		1	
No	5.17 (1.59–16.82)	0.02	1.79 (0.30–10.44)	0.58

## 4. Discussion

### 4.1. Low ART uptake among pregnant women

Our results highlighted low ART uptake among ART-naïve pregnant women who tested positive for HIV and attended ANC in IHCs. Only 34% (29/85) of participants were linked to ART. The ART uptake registered in this study was very low, compared to the national level of uptake in formal healthcare facilities in Cameroon. In 2020, the ART coverage among pregnant women living with HIV was 64% (7).

PWLHIV who were screened in sites that integrated ART in ANC had a better ART uptake [81% (17/21)], compared to those who were referred for ART [18% (12/64)], and this difference was significant (*p* ≤ 0.001). This stipulates that the referral of PWLHIV from an ANC site to a different ART site is a hindrance to ARV uptake in PWLHIV. These findings are consistent with those of a systematic review and meta-analysis, highlighting an increase in ART uptake in clinics that integrated ART in ANC compared to referral-based models [relative risk (RR): 1.37; 95% CI: 1.05–1.79; *I*^2^: 83%] (26). Similar results were reported in a study in Malawi, indicating that non-integration of ART in ANC in the same facility results in the loss of the majority of PWLHIV between ANC and ART clinics. In this study, ART uptake was significantly higher in the model where HIV testing and counseling (HTC) and ANC were fully integrated into ANC compared to the model where HTC was only integrated into ANC services with further referral to an ART clinic for treatment initiation (63% vs. 51%; *p* = 0.001) (27).

This poor linkage to ART following a referral was also reported in a study on Mobile health clinics in South Africa. In this study, ART initiation within 3 months of HIV testing did not reach 50% (28–30).

### 4.2. Acceptable time in ART initiation among initiators

The time between obtaining HIV results and ART initiation was acceptable in our setting, especially for women screened in IHCs integrating ART in ANC, whose median time of initiation was 4 days (IQR, 0–12). Studies carried out in other settings have shown longer delays between HIV diagnosis and ART initiation in PWLHIV of up to 4 weeks (31, 32). The longer delays observed between HIV diagnosis and initiation of ART in these studies could be explained by the prior counseling and education sessions that patients underwent before ART initiation and the non-implementation of Option B+ or test and treat strategy in these settings. However, in some studies implementing Option B+, ART initiation times improved dramatically, with up to 73% of PWLHIV starting ART on the day of receiving their HIV test result (33).

### 4.3. Low ART retention

In this study, we observed a rapid decrease in ART retention in PWLHIV at 3 months post-ART initiation. Retention rates reported at 3 months in other settings were considerably higher than what was recorded in this study. In a retrospective cohort study in Haiti, Puttkammer and colleagues (34) reported a retention rate in ART at 3 months of women in Option B+ at 75%. In addition, a study assessing early retention in ART in rural Zimbabwe reported a retention rate of 84% at 3 months post-ART initiation (35). Moreover, in a systematic review and meta-analysis of studies in Africa, the cumulative retention rate at 3 months after ART initiation in women in Option B+ was 89.9% (36). However, it should be noted that these studies were carried out in formal health facilities. Moreover, the definition of three-month retention we chose in this study may seem very stringent. However, we retained this definition to ensure the women were completely covered during this period, given the need for ART coverage for pregnant women to minimize the risk of HIV transmission to the child, in addition to the low rate of initiation of ANC at the recommended time, i.e., in the first trimester of pregnancy. Nevertheless, the collection of ART is still not always synonymous with treatment adherence, which is an essential parameter too.

A study conducted in Cameroon assessing retention at 12 months post-ART initiation in the general population of people living with HIV showed that the retention of patients on ART was very low. The national average retention rate for patients on ART was 66%. The authors concluded that this reflected the poor compliance of patients with ART (37).

In our study, women diagnosed in IHCs that integrated ART in ANC were more retained in care at 3 months although this result was not significant. Conversely, a study in Malawi showed that retention in ART was higher in women who were referred to other clinics to start ART (27). Meanwhile, another study found retention rates to be similar in both models (26).

### 4.4. Perceived inefficacy and ART non-retention

Our findings indicated that PWLHIV who perceived themselves as unable to initiate ART were more likely to not initiate it. This could be perceived as a lack of self-efficacy to initiate ART. Self-efficacy refers to confidence in the ability to exert control over one’s own motivation, behavior, and social environment (38). Bandura, in an experimental study of the link between perceived self-efficacy and anxiety, showed that perceived inefficacy is accompanied by high levels of subjective distress (39). Similarly, Truong et al. (40), in their study on depression and anxiety, found that depression was associated with 40% lower odds of initiating ART within 3 months of testing positive for HIV (aOR = 0.60, 95% CI = 0.46 to 0.79).

Therefore, our results suggest that pregnant women diagnosed with HIV in IHCs need reinforced HIV counseling and psychological support. These could improve the body of knowledge available, their perception of the disease and the related treatment, and consequently structure their perceived self-efficacy toward ART initiation.

### 4.5. Stigmatization and non-retention in ART

A total of 50% of women who dropped out of ART care reported either stigmatization from health providers encountered in formal treatment centers where they were referred or fear of their partner discovering their status and subsequent stigmatization.

Although decreasing, the stigmatization of people living with HIV remains a reality in Cameroon (41, 42). In a study carried out in South West Cameroon, 44.4% of the 384 participants living with HIV felt ashamed because of their HIV status, and almost 12% were afraid of losing their sexual partners because of their HIV status. The authors concluded that the total index score for external and internal stigmatization was as high as 59.1 (43). This fear of stigma leads to poor linkage and non-retention in PMTCT care (44). Another study in Cameroon reported that 52.8% of pregnant and breastfeeding women living with HIV abandoned ART because of denial of HIV status, stigmatization, and discrimination (42). This stigmatization toward people living with HIV is also experienced within formal health facilities through healthcare providers who are supposed to take care of them. Thus, in Cameroon, the fight against stigmatization and discrimination is a recurrent axis in the national strategic plans for the fight against HIV, AIDS, and sexually transmitted infections (45).

Beyond Cameroon, in a narrative review, Munkhodya and Collaborators (46) highlighted that non-disclosure was the main reason for non-retention in ART. This non-disclosure was created by fear of abandonment, stigmatization, and divorce. In addition, in Dar es Salaam, a study reported poor reception of people living with HIV in health facilities, resulting in the discontinuation of ART. Among these stigmas, the authors mentioned neglect, verbal abuse, excessive use of protective gear like gloves, and less attention to their concerns (47). In a systematic review and meta-analysis of studies on retention in HIV care in Africa, Knettel and colleagues (36) reported stigma and fear of disclosure to the community and intimate partner as barriers to ART enrollment and retention. In addition, the authors identified poor experiences with providers in health facilities as a leading cause of ART dropout. Similar findings were reported in a study in Zambia, assessing factors affecting retention in care (48).

### 4.6. Strategies to improve uptake and retention in care

Some authors recommended establishing strong community systems for monitoring and supporting patients on ART to improve retention. A wider range of partners, such as community organizations and, in particular, those of people living with HIV should be considered for improving enrollment and retention in ART (48, 49).

Besides this psychosocial support from lay workers and associations, the support of partners is also crucial. This partner support begins with their involvement in antenatal HIV counseling and testing. Two studies conducted in Cameroon demonstrated that administrating a new couple-oriented antenatal HIV counseling could improve the frequency of HIV testing among male partners. Indeed, this frequency improved to 27% in urban areas and 18% in rural areas. Additionally, better PMTCT outcomes were recorded in women whose partners were involved (50).

In a systematic review, Vrazo and collaborators (51) reviewed a set of interventions that significantly improved ART enrolment and retention in the PMTCT continuum of care. They concluded that interventions, including ANC/ART integration, family-centered approaches, and the use of lay healthcare providers, are demonstrably effective in increasing service uptake and retention of HIV-positive mothers and their infants in PMTCT programs. The same results were highlighted in another systematic review synthesizing evidence on the effectiveness of interventions to improve uptake and retention in PMTCT (52). However, the authors noted that overall findings were mixed and effect sizes were small.

### 4.7. Implication for the Cameroonian health system

Many of these strategies are being implemented in Cameroon, particularly the integration of PMTCT into maternal, neonatal, and child health in the majority of health facilities; the family approach to HIV care, including the involvement of male partners; the delegation of tasks, which allows decentralization of ART; community distribution of ART; and experimentation of the User fees policy. However, IHCs are not reached by all these strategies due to their illegal status.

Our results showed that nearly 36% (33/92) of the participants who did not initiate ART gave as a reason the fear of the reception in the ART-dispensing centers where they were referred. These ART-dispensing centers were mostly public health facilities. This is understandable since public health facilities in Cameroon have acquired a bad reputation over time linked to recurrent poor practices and dysfunctions (24, 25). These have made them places of conflict between health workers and patients (53).

Due to the limited duration of PMTCT follow-up in IHCs, poorly established ART supply networks, and lack of supervision and monitoring of these IHCs by the National PMTCT program, a process of integration within the formal care system of PWLVIH who are screened in IHCs should be considered. Active associations of people living with HIV may be used as bridges between IHCs and formal HIV care centers for this purpose. Alongside this intervention, it would be critical to consider health policies aimed at improving public health facilities. In addition, simplifying the procedures for opening private health centers could help reduce the extent of operating IHCs.

### 4.8. Strengths of the study

This is the first time that a documented study on PMTCT in IHCs has been conducted on informal health facilities and provides information that subsequent studies could build on for comparisons or in-depth investigations.

### 4.9. Limitations

As a limitation of this study, it is likely that the period considered to assess ART enrollment (3 months after HIV test results) has underestimated the true number of women who eventually initiated ART. However, since people living with HIV should start ART within 2 weeks of HIV diagnosis according to the national HIV guidelines in Cameroon, we went up to 3 months to consider late initiators.

The phenomenon of IHCs offering ANC services is widespread in all regions of the country, and we only conducted our study in two cities. Therefore, it is important that additional research in this area be carried out in other regions of the country to estimate the extent of the situation within the country.

Moreover, due to the small sample size, other important factors of non-retention in ART may not have been detected.

## 5. Conclusion

Enrolment of PWLHIV in ART within 3 months after HIV testing and retention in ART 3 months post-initiation were low in our setting. The low perceived self-efficacy to initiate ART after HIV testing was associated with ART non-retention in ART. At a time when the National PMTCT Program makes the residual elimination of mother-to-child transmission of HIV a major challenge to achieve, the enrolment of PWLHIV in ART and their retention in the continuum of PMTCT care are essential to prevent jeopardizing all the efforts for PMTCT made so far. This will help to achieve the 95-95-95 UNAIDS goal. Thus, the poor outcomes of pregnant women diagnosed with HIV in IHCs during ANC highlighted in this study should draw the attention of the PMTCT Program, in particular, and the overall Public Health policy in Cameroon.

## Data availability statement

The datasets presented in this study can be found in online repositories. The names of the repository/repositories and accession number(s) can be found at: Dryad Digital Repository. Available at https://datadryad.org/stash/share/gVd586V9mLAcoNBvsWcvXcEY6pPi5Ik5k_gPiYeImL8.

## Ethics statement

The study involving human participants was reviewed and approved by the Cameroon National Ethics Committee for Research in Human Health and Cameroonian Ministry of Public Health. The patients/participants provided their written informed consent to participate in this study. Written informed consent was obtained from the individual(s) for the publication of any potentially identifiable images or data included in this article.

## Author contributions

AASO designed the study. AASO and AAS cleaned the database and drafted the manuscript. AASO and FY participated in the data analysis and interpretation. All authors contributed to the article and approved the submitted version.

## Conflict of interest

The authors declare that the research was conducted in the absence of any commercial or financial relationships that could be construed as a potential conflict of interest.

## Publisher’s note

All claims expressed in this article are solely those of the authors and do not necessarily represent those of their affiliated organizations, or those of the publisher, the editors and the reviewers. Any product that may be evaluated in this article, or claim that may be made by its manufacturer, is not guaranteed or endorsed by the publisher.

## References

[1] De Cock KM Fowler MG Mercier E de Vincenzi I Saba J Hoff E. Prevention of mother-to-child HIV transmission in resource-poor countries: translating research into policy and practice. JAMA. (2000) 283:1175–82. doi: 10.1001/jama.283.9.1175 10703780

[2] Embree J. The impact of HIV/AIDS on children in developing countries. Paediatr Child Health. (2005) 10:261–3. 19668627 PMC2722540

[3] Gong T Wang H He X Liu J Wu Q Wang J. Investigation of prevention of mother to child HIV transmission program from 2011 to 2017 in Suzhou, China. Sci Rep. (2018) 8:18071. doi: 10.1038/s41598-018-36623-6, PMID: 30584264 PMC6305485

[4] Kassa GM. Mother-to-child transmission of HIV infection and its associated factors in Ethiopia: a systematic review and meta-analysis. BMC Infect Dis. (2018) 18:216. doi: 10.1186/s12879-018-3126-5, PMID: 29747581 PMC5946547

[5] Tudor Car L Van Velthoven MHMMT Brusamento S Elmoniry H Car J Majeed A. Integrating prevention of mother-to-child HIV transmission programs to improve uptake: a systematic review. PLoS One. (2012) 7:e35268. doi: 10.1371/journal.pone.0035268, PMID: 22558134 PMC3338706

[6] UNAIDS. UNAIDS data 2021. Geneva, Switzerland: UNAIDS (2021). 2021 p.

[7] UNAIDS. The state of HIV prevention in Cameroon In: Epidemiological estimates; global AIDS monitoring. Geneva, Switzerland: UNAIDS (2021).

[8] Ahoua L Arikawa S Tiendrebeogo T Lahuerta M Aly D Becquet R. Measuring retention in care for HIV-positive pregnant women in prevention of mother-to-child transmission of HIV (PMTCT) option B+ programs: the Mozambique experience. BMC Public Health. (2020) 20:322. doi: 10.1186/s12889-020-8406-5 32164601 PMC7069209

[9] UNICEF. Evidence-based practices for retention in care of mother-infant pairs in the context of eliminating mother-to-child transmission of HIV in eastern and southern Africa. Geneva, Switzerland: UNICEF (2019).

[10] Ministère de la Santé Publique. Prise en charge des personnes vivant avec le VIH au Cameroun. Yaounde: Ministry of Public Health (2016).

[11] World Health Organization Consolidated guidelines on the use of antiretroviral drugs for treating and preventing HIV infection: recommendations for a public health approach. Geneva, Switzerland: WHO (2013). 24716260

[12] Tweya H Gugsa S Hosseinipour M Speight C Ng’ambi W Bokosi M. Understanding factors, outcomes and reasons for loss to follow-up among women in option B+ PMTCT programme in Lilongwe, Malawi. Trop Med Int Health. (2014) 19:1360–6. doi: 10.1111/tmi.12369, PMID: 25087778

[13] Vogt F Ferreyra C Bernasconi A Ncube L Taziwa F Marange W. Tracing defaulters in HIV prevention of mother-to-child transmission programmes through community health workers: results from a rural setting in Zimbabwe. J Int AIDS Soc. (2015) 18:20022. doi: 10.7448/IAS.18.1.20022, PMID: 26462714 PMC4604210

[14] CNLS. Rapport de progrès PTME No. 12 In: ANNEE 2017. Yaounde, Cameroun: CNLS (2018)

[15] Van der Geest S. Les médicaments sur un marché camerounais: Reconsidération de la commodification et de la pharmaceuticalisation de la santé. Anthropologiesante. (2017) 14 doi: 10.4000/anthropologiesante.2450

[16] Meva’a Abomo D. Le fardeau de la lutte contre le paludisme urbain au Cameroun: état des lieux, contraintes et perspectives. Revue Canadienne de Géographie Tropicale. (2016) 3:26–42.

[17] Socpa A. (1995) Les Pharmacies de rue dans l’espace médical urbain. Emergence et déterminants des stratégies informelles d’accès aux médicaments à Douala. Anthropologie. Yaoundé, Cameroun: Université de Yaoundé I.

[18] Wogaing J. De la quête à la consommation du médicament au Cameroun. Revue Internationale sur le Médicament. (2010):3.

[19] Institut National de la Statistique. Troisième Enquête Camerounaise auprès des Ménages 2007 (2007). Available at: https://www.ilo.org/surveyLib/index.php/catalog/374 (Accessed March 6, 2021).

[20] Mendo E. Les micro-unités informelles de santé au Cameroun. Paris: L’Harmattan (2018). 412 p.

[21] Ministry of Public Health. Assainissement de la carte sanitaire. Yaounde, Cameroun: Ministry of Public Health (2017).

[22] CNLS. Rapport progrès PTME 2019. Yaoundé, Cameroun: CNLS (2020).

[23] Ministry of Health. Health sector strategy 2016–2027_Cameroon. Yaounde, Cameroon: Ministry of Public Health (2015).

[24] Ministère de la Santé Publique. Formations sanitaires. Les directives sur l’acceuil des patients. Yaounde, Cameroun: Ministry of Public Health (2016).

[25] NGA NKOUMA TSANGA RC. Effets de la corruption en milieu hospitalier camerounais sur la performance hospitalière. Repères et Perspectives Economiques. (2020):4.

[26] Suthar AB Hoos D Beqiri A Lorenz-Dehne K McClure C Duncombe C. Integrating antiretroviral therapy into antenatal care and maternal and child health settings: a systematic review and meta-analysis. Bull World Health Organ. (2013) 91:46–56. doi: 10.2471/BLT.12.107003, PMID: 23397350 PMC3537248

[27] Chan AK Kanike E Bedell R Mayuni I Manyera R Mlotha W. Same day HIV diagnosis and antiretroviral therapy initiation affects retention in option B+ prevention of mother-to-child transmission services at antenatal care in Zomba District, Malawi. J Int AIDS Soc. (2016) 19:20672. doi: 10.7448/IAS.19.1.20672, PMID: 26976377 PMC4789547

[28] Labhardt ND Motlomelo M Cerutti B Pfeiffer K Kamele M Hobbins MA. Home-based versus mobile clinic HIV testing and counseling in rural Lesotho: a cluster-randomized trial. PLoS Med. (2014) 11:e1001768. doi: 10.1371/journal.pmed.1001768 25513807 PMC4267810

[29] Maughan-Brown B Harrison A Galárraga O Kuo C Smith P Bekker L-G. Factors affecting linkage to HIV care and ART initiation following referral for ART by a mobile health clinic in South Africa: evidence from a multimethod study. J Behav Med. (2019) 42:883–97. doi: 10.1007/s10865-018-0005-x, PMID: 30635862 PMC6625943

[30] Bassett IV Regan S Luthuli P Mbonambi H Bearnot B Pendleton A. Linkage to care following community-based mobile HIV testing compared with clinic-based testing in Umlazi township, Durban, South Africa. HIV Med. (2014) 15:367–72. doi: 10.1111/hiv.12115, PMID: 24251725 PMC4026348

[31] Kyaw KWY Oo MM Kyaw NTT Phyo KH Aung TK Mya T. Low mother-to-child HIV transmission rate but high loss-to-follow-up among mothers and babies in Mandalay, Myanmar; a cohort study. PLoS One. (2017) 12:e0184426. doi: 10.1371/journal.pone.0184426 28886165 PMC5590939

[32] Myer L Zulliger R Bekker L-G Abrams E. Systemic delays in the initiation of antiretroviral therapy during pregnancy do not improve outcomes of HIV-positive mothers: a cohort study. BMC Pregnancy Childbirth. (2012) 12:94. doi: 10.1186/1471-2393-12-94 22963318 PMC3490939

[33] Langwenya N Phillips TK Brittain K Zerbe A Abrams EJ Myer L. Same-day antiretroviral therapy (ART) initiation in pregnancy is not associated with viral suppression or engagement in care: a cohort study. J Int AIDS Soc. (2018) 21:e25133. doi: 10.1002/jia2.25133, PMID: 29939483 PMC6016637

[34] Puttkammer N Domerçant JW Adler M Yuhas K Myrtil M Young P. ART attrition and risk factors among option B+ patients in Haiti: a retrospective cohort study. PLoS One. (2017) 12:e0173123. doi: 10.1371/journal.pone.0173123, PMID: 28264045 PMC5338795

[35] Chimwaza AN Tweya H Mugurungi O Mushavi A Mukungunugwa S Sithole N. Early retention among pregnant women on ‘option B +’ in urban and rural Zimbabwe. AIDS Res Ther. (2021) 18:10. doi: 10.1186/s12981-021-00333-3, PMID: 33794957 PMC8015197

[36] Knettel BA Cichowitz C Ngocho JS Knippler ET Chumba LN Mmbaga BT. Retention in HIV care during pregnancy and the postpartum period in the option B+ era: a systematic review and meta-analysis of studies in Africa. J Acquir Immune Defic Syndr. (2018) 77:427–38. doi: 10.1097/QAI.0000000000001616, PMID: 29287029 PMC5844830

[37] Billong SC Penda CI Fokam J Mvilongo AE Fodjo R Messeh A. Profil national des indicateurs d’alerte précoce de la pharmaco-résistance du VIH au Cameroun. Pan Afr Med J. (2020) 37:374. doi: 10.11604/pamj.2020.37.374.17649, PMID: 33796187 PMC7992409

[38] Bandura A. Self-efficacy: the exercise of control. W. H. Freeman/times books. New York, NY: Henry Holt & Co. (1997).

[39] Bandura A. Self-efficacy conception of anxiety. Anxiety Res. (1988) 1:77–98. doi: 10.1080/10615808808248222

[40] Truong M Rane MS Govere S Galagan SR Moosa M-Y Stoep AV. Depression and anxiety as barriers to art initiation, retention in care, and treatment outcomes in KwaZulu-Natal, South Africa. EClinicalMedicine. (2021) 31:100621. doi: 10.1016/j.eclinm.2020.100621, PMID: 33490927 PMC7806795

[41] The Global Fund. Baseline assessment—Cameroon scaling up programs to reduce human rights-related barriers to HIV and TB services. Geneva, Switzerland: UNAIDS (2018).

[42] Atanga PN. Retention-in-care, adherence and treatment outcomes in a cohort of HIVpositive pregnant and breastfeeding women enrolled in a pilot project implementing “option B+”. Cameroon. Munich: Medical Centre of the University of Munich (2017). 167 p.

[43] Egbe TO Nge CA Ngouekam H Asonganyi E Nsagha DS. Stigmatization among people living with HIV/AIDS at the Kumba Health District, Cameroon. J Int Assoc Provid AIDS Care. (2020) 19:232595821989930. doi: 10.1177/2325958219899305, PMID: 31908184 PMC6947670

[44] Alhassan Y Twimukye A Malaba T Myer L Waitt C Lamorde M. “I fear my partner will abandon me”: the intersection of late initiation of antenatal care in pregnancy and poor ART adherence among women living with HIV in South Africa and Uganda. BMC Pregnancy Childbirth. (2022) 22:566. doi: 10.1186/s12884-022-04896-5, PMID: 35840939 PMC9284724

[45] National AIDS Control Committee. Cameroon National Strategic Plan for fight against HIV/AIDS and STIs 2021–2023. Yaounde, Cameroun: (2021).

[46] Mbeya Munkhondya TE Smyth RM Lavender T. Facilitators and barriers to retention in care under universal antiretroviral therapy (option B+) for the prevention of mother to child transmission of HIV (PMTCT): a narrative review. Int J Africa Nurs Sci. (2021) 15:100372. doi: 10.1016/j.ijans.2021.100372

[47] Mhode M Nyamhanga T. Experiences and impact of stigma and discrimination among people on antiretroviral therapy in Dar Es Salaam: a qualitative perspective. AIDS Res Treat. (2016) 2016:1–11. doi: 10.1155/2016/7925052, PMID: 27110395 PMC4823479

[48] Mukumbang FC Mwale JC van Wyk B. Conceptualising the factors affecting retention in care of patients on antiretroviral treatment in Kabwe District, Zambia, using the ecological framework. AIDS Res Treat. (2017) 2017:1–11. doi: 10.1155/2017/7356362, PMID: 29250442 PMC5700481

[49] Ware NC Wyatt MA Geng EH Kaaya SF Agbaji OO Muyindike WR. Toward an understanding of disengagement from HIV treatment and care in sub-Saharan Africa: a qualitative study. PLoS Med. (2013) 10:e1001369:e1001369; discussion e1001369. doi: 10.1371/journal.pmed.1001369, PMID: 23341753 PMC3541407

[50] Tchendjou Tankam PY. Conseil prénatal du VIH orienté vers le couple: faisabilité et effets sur la prévention du VIH au Cameroun. Bordeaux, France: Université de Bordeaux (2014).

[51] Vrazo AC Firth J Amzel A Sedillo R Ryan J Phelps BR. Interventions to significantly improve service uptake and retention of HIV-positive pregnant women and HIV-exposed infants along the prevention of mother-to-child transmission continuum of care: systematic review. Tropical Med Int Health. (2018) 23:136–48. doi: 10.1111/tmi.13014, PMID: 29164754

[52] Ritchie LMP van Lettow M Pham B Straus SE Hosseinipour MC Rosenberg NE. What interventions are effective in improving uptake and retention of HIV-positive pregnant and breastfeeding women and their infants in prevention of mother to child transmission care programmes in low-income and middle-income countries? A systematic review and meta-analysis. BMJ Open. (2019) 9:e024907. doi: 10.1136/bmjopen-2018-024907, PMID: 31362959 PMC6677958

[53] Socpa A. Djouda Feudjio YB. "L’hôpital au Cameroun: lieu de soins ou espace conflictuel?" In: Nkoum BA, editor. Santé plurielle en Afrique. Perspective pluridisciplinaire: Paris, France L’Harmattan (2011). 337–59.

